# Personalisation of the management of schizophrenia and other primary psychoses

**DOI:** 10.1192/j.eurpsy.2023.164

**Published:** 2023-07-19

**Authors:** S. Galderisi

**Affiliations:** Psychiatry, University of Campania Luigi Vanvitelli, Napoli, Italy

## Abstract

**Abstract:**

In the treatment of persons with schizophrenia the goal has gradually shifted from the reduction of symptoms and prevention of relapse to recovery. However, this goal is achieved for a minority of persons with schizophrenia, while for most of them the disorder still is a major cause of disability, poor quality of life and premature death, and presents considerable social and economic costs.

Studies aimed at identifying variables with a significant impact on schizophrenia outcome indicate that early intervention, shared decision making, treatment continuity, physical comorbidities, negative symptoms, deficits in cognitive functions and functional capacity account for most of the functional impairment of patients but are often neglected in current clinical practice.

In this presentation, I will illustrate the role of these variables and the need for an in-depth clinical characterization of persons with primary psychoses to implement personalized treatment plans and improve the care of people with schizophrenia.

**Disclosure of Interest:**

None Declared

